# Impact of Revascularization on Major Adverse Cardiovascular Events in Patients Without ST-Elevation Myocardial Infarction in the Arabian Gulf

**DOI:** 10.3390/jcdd12040117

**Published:** 2025-03-27

**Authors:** Ibrahim Al-Zakwani, Fahad AlKindi, Wael Almahmeed, Mohammad Zubaid

**Affiliations:** 1 Department of Pharmacology & Clinical Pharmacy, College of Medicine & Health Sciences, Sultan Qaboos University, Muscat 123, Oman; 2 Sultan Qaboos University Hospital, University Medical City, Muscat 123, Oman; f.alkindi@squ.edu.om; 3 Department of Medicine, College of Medicine & Health Sciences, Sultan Qaboos University, Muscat 123, Oman; 4 Heart & Vascular Institute, Cleveland Clinic Abu Dhabi, Abu Dhabi P.O. Box 112412, United Arab Emirates; mahmeew@clevelandclinicabudhabi.ae; 5 Department of Medicine, Faculty of Medicine, Kuwait University, Kuwait City 13110, Kuwait; mohammed.zubaid@ku.edu.kw

**Keywords:** percutaneous coronary intervention, coronary artery bypass graft, acute coronary syndrome, stroke, myocardial infarction, mortality, hospital readmission, Middle East

## Abstract

**Objectives**: The study evaluated the impact of revascularization procedures, including percutaneous coronary intervention (PCI) and coronary artery bypass graft (CABG), during the index hospitalization on major adverse cardiovascular events (MACE) in patients with non-ST-elevation myocardial infarction (NSTEMI) in the Arabian Gulf. **Methods**: Data were analyzed from 1820 consecutive patients diagnosed with NSTEMI, admitted to 29 hospitals in four Arabian Gulf countries from January 2012 to January 2013, and who were discharged alive. **Results**: Of the patients, 29.1% (n = 529) underwent either PCI (89.8%; n = 475) or CABG (10.2%; n = 54). The matching method (288 PCI/CABG patients were matched with 762 controls that did not undergo PCI/CABG) revealed significant reductions in MACE events among patients who had undergone PCI/CABG (25% vs. 43%; *p* < 0.001). This decrease was consistent across individual MACE components, including stroke/transient ischemic attack (TIA) (2.4% vs. 7.0%; *p* = 0.005), all-cause mortality (4.5% vs. 7.0%; *p* < 0.001) and cardiac-related readmissions (20% vs. 31%; *p* = 0.001) but not reinfarction (1.7% vs. 1.4%; *p* = 0.73). **Conclusions**: The revascularization procedures, PCI/CABG, were associated with significant reductions in annual MACE event rates, specifically lower stroke/TIA, all-cause mortality and cardiac-related readmissions.

## 1. Introduction

Acute coronary syndromes (ACS), including non-ST-elevation myocardial infarction (NSTEMI), are one of the leading causes of mortality in the Asia Pacific region, accounting for nearly half of the global burden of the disease [1]. ACS is also associated with significant morbidity, with nearly 130 million disability-adjusted life years (DALYs) reported annually [2]. Revascularization therapy (especially in high-risk patients), including percutaneous coronary intervention (PCI) and coronary artery bypass graft (CABG), along with pharmacological treatment, have been the mainstay in the management of NSTEMI ACS as recommended by the European Society of Cardiology (ESC) [3] and the American Heart Association (AHA)/American College of Cardiology (ACC) Task Force on Clinical Practice Guidelines [4].

The benefits of PCI/CABG in the reduction of major adverse cardiovascular events (MACE) are well established [5,6,7]. However, health disparities by race, ethnicity, access to health care, hospital capability and quality, health education, socioeconomic status, and insurance access have been associated with adverse cardiovascular health outcomes [8,9,10,11,12]. Furthermore, data on the benefits of PCI/CABG among patients with NSTEMI in the Arabian Gulf region are limited, with the only pertinent published study evaluating mortality and none of the other MACE components [13]. The Gulf COAST was a prospective, multi-country, multicenter registry of consecutive Arabian Gulf citizens hospitalized with the diagnosis of ACS in four Arabian Gulf countries [14]. This registry provides a unique opportunity to evaluate the impact of revascularization therapy on cardiovascular outcomes among ACS patients in the region. Hence, the aim of this study was to evaluate the impact of PCI/CABG at index hospitalization on MACE events in patients with NSTEMI in the Arabian Gulf.

## 2. Methods

### 2.1. Study Design, Setting, and Population

Details of the methods of the Gulf COAST registry have already been previously reported [14]. Briefly, Gulf COAST (Gulf locals with acute coronary syndrome events) registry was a prospective, multicenter, multinational, longitudinal, cohort study of consecutive citizens from four countries in the Arabian Gulf (Bahrain, Kuwait, Oman, and United Arab Emirates) admitted from January 2012 to January 2013 to 29 hospitals with a diagnosis of ACS. However, the analyses of this study were retrospective in design. The registry enrolled a total of 4044 patients who were 18 years of age or older with ACS diagnosed according to American College of Cardiology (ACC) clinical data standards [15]. Specifically, stroke (or transient ischemic attack (TIA)) was defined as any loss of neurological function caused by an ischemic or hemorrhagic event, with residual symptoms at least 24 h after onset or leading to death, and diagnosed with the aid of imaging data (CT scan or MRI) and lumbar puncture. Reinfarction was any acute myocardial infarction, cardiac-related readmission was defined as any readmission associated with cardiovascular disease, while mortality was defined as any death, regardless of etiology, during the follow-up period after the index hospital stay. Apart from excluding non-citizens and those who were not willing/able to provide consent, there were no other exclusion criteria.

The revascularization procedures (PCI/CABG) were those that were performed during the index hospital admission. MACE events included stroke/TIA, reinfarction, all-cause mortality, and cardiac-related readmissions that were captured during the 12-months follow-up after hospital discharge from the index hospital admission.

Data collected included patient demographics, previous medical and cardiovascular disease risk factors, prior medication use, laboratory data, clinical presentation, and management during hospital stay, including medications, reperfusion therapy, and procedures, as well as discharge medications. Follow-up was performed at 12 months post hospital discharge and was carried out by clinic visits or telephone interviews.

### 2.2. Ethical Approval

The study was approved by the local institutional ethics committees of participating centers in the four Arabian Gulf countries (Kuwait, Joint Committee for the Protection of Human Subjects in Research, VDR/JC/89, 13 October 2011; Oman, Ethical Review and Approval Committee, MH/DGP/R&S/PROPOSAL_APPROVED/1/2012, 9 January 2012; Bahrain, Secondary Care Medical Research Subcommittee, Ministry of Health, 23 December 2011; Abu Dhabi UAE, Institutional Review Board/Research Ethics Committee, Sheikh Khalifa Medical City, REC-24.11.2011 [RS 189], 24 November 2011; Abu Dhabi UAE, Institutional Review Board, Medical Services Corps, General Head Quarters Armed Forces, 18 November 2011; Al Ain UAE, Al Ain Medical District Human Research Ethics Committee, Faculty of Medicine and Health Sciences, Posted: 25 July 2023 doi:10.20944/preprints202307.1647.v1 3 Emirates University, Protocol No. 11/48, 24 November 2011; Dubai UAE, Medical Research Committee, Dubai Health Authority, MRC-11/2011_2, 30 November 2011).

### 2.3. Statistical Analysis

For categorical variables, frequencies and percentages were reported. Differences between groups were analyzed using Pearson’s χ^2^ tests (or Fisher’s exact tests for expected cells of <5). For continuous variables, mean and standard deviation were used to present the data, while analyses were performed using Student’s t-test. Continuous variables that were not normally distributed were summarized using median and interquartile range and analyses conducted using Wilcoxon–Mann–Whitney tests [16].

The impact of PCI/CABG on MACE events (including stroke/TIA, all-cause mortality, reinfarction, and cardiac-related readmissions) at 1-year follow-up after the index hospitalization were evaluated using the entropy-balancing (EB) matching method, which included the following variables in its models: age; gender; body mass index (BMI); smoking status; Global Registry of Acute Coronary Events (GRACE) risk score; atrial fibrillation; troponin levels during the index admission; diabetes mellitus; hypertension; dyslipidemia; Killip class; left ventricular ejection fraction; cerebrovascular disease (stroke/TIA, coronary artery disease, peripheral vascular disease); cardiogenic shock during the index admission; chronic renal failure; prior PCI/CABG; low molecular weight heparin/unfractionated heparin, glycoprotein IIb/IIIa inhibitors, thrombolytics, inotropes during the index admission; aspirin, platelet inhibitors, renin–angiotensin-system blockers, beta blockers, and statins during discharge. Absolute standardized mean differences (SMDs) above 0.1 (10%) or 0.25 (25%) are indicative of covariate imbalance, as suggested by Normand and colleagues [17] and Ruben [18], respectively. An a priori two-tailed level of significance was set at the 0.05 level. Statistical analyses were conducted using STATA version 16.1 (StataCorp, 2013, Stata Statistical Software, College Station, TX, USA).

## 3. Results

**Figure 1:**
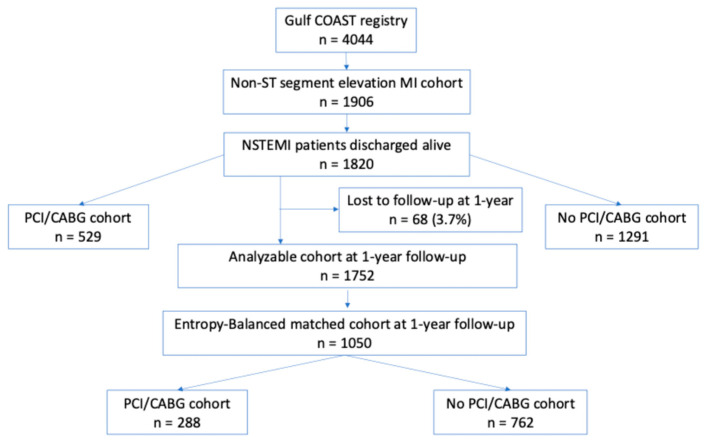
Schematic flow chart of the original Gulf COAST NSTEMI ACS cohort. NSTEMI, non-ST-elevation myocardial infarction; ACS, acute coronary syndrome; MI, myocardial infarction; PCI, percutaneous coronary intervention; CABG, coronary artery bypass graft.

As illustrated in Figure 1, out of the 4044 patients recorded in the Gulf COAST registry, we utilized the data of only 1820 patients who had NSTEMI and were consequently discharged alive from the hospital for this analysis. Of these, a total of 29.1% (n = 529) had either PCI (89.8% n = 475) or CABG (10.2%; n = 54) during the index hospital admission. Of note, out of the original cohort of 1291 NSTEMI patients who did not undergo PCI/CABG, 18.7% (242/1291) were catheterized or had cardiac CT during the index hospital stay. The overall mean age of the cohort was 61.7 ± 12.2 years, and 65.4% (n = 1190) were males. A total of 20.7% (n = 377) of the patients were current smokers. The three most prevalent comorbid conditions among this cohort were hypertension (n = 1283; 70.5%), dyslipidemia (n = 1087; 59.7%), and diabetes mellitus (n = 1048; 57.6%). At 1-year follow-up, 3.7% (n = 68) of the patients were lost to follow-up.

As shown in Table 1, patients who had undergone PCI/CABG were younger, had lower GRACE risk scores, were more likely to be male, smokers, or dyslipidemic, had in-hospital cardiogenic shock, and had prior PCI/CABG before the index admission. However, they were less likely to have atrial fibrillation, higher Killip class (II/III/IV), and low LVEF (<40%) when compared to those that did not undergo PCI/CABG. During the index admission, patients that had PCI/CABG were more likely to be prescribed glycoprotein IIb/IIIa inhibitors and inotropes. They were also more likely to be discharged with aspirin, platelet inhibitors, beta-blockers, and statins, compared to those who did not undergo PCI/CABG. Table 1 also outlines the covariate balance across the groups before and after the EB matching method. The EB matching method led to the significant reduction in the overall mean and median bias of 1.2% (from 20.5%) and 0.6% (from 19.7%), respectively. There were largely no significant differences among the groups with regards to the demographic and clinical characteristics after matching, as demonstrated by the insignificant differences of covariate imbalance (low SMDs of <10%) and the non-significant *p*-values (*p* > 0.05).

Table 2 presents the benefits of PCI/CABG on MACE events that were captured during the one-year period post-discharge after the index admission. At 12 months follow-up, the EB matching method, in which 288 PCI/CABG patients were matched with 762 controls who did not undergo PCI/CABG, revealed significant reductions in MACE events among patients who had undergone PCI/CABG (25% vs. 43%; *p* < 0.001). This decrease was consistent across individual MACE components, including stroke/TIA (2.4% vs. 7.0%; *p* = 0.005), all-cause mortality (4.5% vs. 13%; *p* < 0.001), and cardiac-related readmissions (20% vs. 31%; *p* = 0.001) but not for reinfarction (1.7% vs. 1.4%; *p* = 0.73).

## 4. Discussion

This large, multinational, multicenter study from the Arabian Gulf region demonstrates that, at 12 months follow-up, NSTEMI patients who had undergone PCI/CABG at index hospitalization were 17.4% significantly less likely to experience MACE events compared to patients who did not undergo PCI/CABG. Specifically, after the EB matching method, NSTEMI patients were 3.8% significantly less likely to suffer stroke/TIA, 6.4% less likely to die, and 11.2% less likely to be readmitted for any cardiac reason.

Our observations largely align with the outcomes of a meta-analysis encompassing seven randomized clinical trials, involving 8375 patients diagnosed with NSTEMI [5]. This meta-analysis demonstrated that the adoption of an early invasive strategy correlated with reduced incidences of 2-year all-cause mortality (4.9% vs. 6.5%), 2-year nonfatal MI (7.6% vs. 9.1%), and 13-month rehospitalization for recurrent ACS events (19.9% vs. 28.7%) [5]. The contrasting differences in the reduction of reinfarction rates between this meta-analysis and the current study could be due to the differences in follow-up periods between the two studies (2 vs. 1 year). It has been reported that longer follow-up periods are associated with increased rates of reinfarction, and this could have contributed to the non-significant results in the current study [19].

In the Arabian Gulf region, registry data derived from six countries further substantiated the superiority of PCI and CABG over conservative management in NSTEMI cases. The PCI group exhibited significantly superior unadjusted and adjusted in-hospital, 30-day, and 1-year mortality rates in comparison to the conservative management group [13]. These findings are similar to those observed in the current study. However, the current study, in addition to mortality, also evaluated stroke/TIA, reinfarction, and cardiac-related readmission.

While trials such as TIMACS [20] and VERDICT [21] have emphasized the benefits of early invasive strategies in individuals with GRACE scores surpassing 140, our investigation indicates that even those with mean scores around 130 exhibit improved survival outcomes. This observation may be attributed to our cohort’s higher incidence of in-hospital cardiogenic shock, a greater prevalence of prior PCI/CABG history before the index admission, and a higher prescription rate of inotropes. These findings resonate with a multinational, multicenter prospective registry comprising of 5856 NSTEMI patients, stratified based on symptom-to-catheter (StC) time. The registry elucidated a reduced risk of all-cause mortality associated with the early implementation of invasive strategies, even among patients with low GRACE risk scores, where the average score in their cohort was 117, which is even lower than in the present study [22].

Within our study cohort, only 29.1% of the patients underwent revascularization, with the majority (89.8%) opting for PCI and only 10.2% for CABG. The suboptimal utilization of invasive treatments prompts inquiries into factors such as physicians underestimating long-term mortality risk in NSTEMI, procedural availability, and healthcare system policies, necessitating further exploration and consideration [23,24,25].

Myocardial viability is critical in determining the long-term outcomes after an ACS event. The extent of viable myocardial tissue left after the infarction directly impacts the heart’s functional recovery, prognosis, and risk of future adverse events. Preserving viable myocardium allows for better cardiac remodeling and improved ventricular function, reducing the likelihood of heart failure, arrhythmias, or recurrent infarctions. Assessing myocardial viability through imaging techniques helps guide therapeutic decisions, such as revascularization, to improve survival and quality of life post-myocardial infarction. Thus, understanding and preserving myocardial viability are key factors in managing and mitigating the future risks following an acute myocardial infarction [26].

Our study has limitations. Due to its inherent nature, observational studies are limited in their abilities to assess causal relationships. Second, all-cause mortality was reported, whereas, if cardiovascular mortality was available, it would have been more pertinent. Third, it would have been more informative if the type of stroke (ischemic/hemorrhagic) had also been recorded. Fourth, a total of 3.7% (n = 68) of the patients were lost to follow-up at 1 year. However, this number was relatively small, and, importantly, there were largely no significant differences in the demographic and clinical and characteristics of the NSTEMI group lost to follow-up at 1 year against the cohort that remained at the end of the year except for smoking status and aspirin (Table 3). It is important to note that covariate imbalances were eventually corrected by the EB matching method, as shown in Table 1. Fifth, the lack of detailed procedural and angiographic as well as imaging data is also a limitation. Additionally, data regarding periprocedural myocardial infarction, which substantially increases short- and long-term mortality risk in NSTEMI patients, are also lacking [27]. Finally, it could have been more appropriate to construct Kaplan–Mier curves of these events; however, the Gulf COAST registry did not capture the timing of the events, only whether events occurred or not.

## 5. Conclusions

To our knowledge, this is one of only a few studies in the Arabian Gulf region to have reported the benefits of PCI/CABG on MACE events after the index hospitalization in NSTEMI patients. At 12 months follow-up after an initial index ACS event, in-hospital PCI/CABG was associated with significantly lower MACE events, specifically stroke/TIA, all-cause mortality, and cardiac-related readmissions among Arabian Gulf citizens. However, PCI/CABG was not associated with any significant differences in reinfarction rates.

## Figures and Tables

**Table 1:** Demographic and clinical characteristics.

Characteristic	Original Unmatched Cohort (n = 1820)					EB Matched Cohort (n = 1050)				
	PCI/CABG					PCI/CABG				
	All (n = 1820)	With (n = 529)	Without (n = 1291)	*p*-Value	SMD	All (n = 1050)	With (n = 288)	Without (n = 762)	*p*-Value	SMD
**Demographic,** n (%) unless stated otherwise										
Age, years	61.7	59.7	62.5	<0.001	−19.7 *	62.2	60.4	60.5	0.954	−0.5
Male gender, n (%)	65.4	71.5	62.9	<0.001	19.8 *	64.9	72.0	71.9	0.982	0.2
BMI, kg/m^2^	28.5	28.5	28.5	0.916	0	28.7	28.7	28.7	0.916	0.9
Smoker, n (%)	20.7	25.0	19.0	0.004	19.1 *	20.0	26.7	25.3	0.712	3.2
**Clinical,** n (%) unless stated otherwise										
GRACE risk score	132.1	123.6	135.6	<0.001	−25.6 *	127.7	127.8	127.7	0.956	0.4
Atrial fibrillation	4.6	2.1	5.7	0.005	−21.2 *	2.1	2.0	2.0	0.962	−0.3
Trop, ng/mL	26.5	35.5	22.9	0.018	9.4	30.7	29.9	30.7	0.937	−0.7
Diabetes mellitus	57.6	55.2	58.6	0.188	−2.0	55.9	56.8	55.9	0.836	1.7
Hypertension	70.5	68.2	71.4	0.177	0.9	71.9	72.0	71.9	0.982	0.2
Dyslipidemia	59.7	69.9	55.5	<0.001	28.0 *	67.8	68.6	67.7	0.821	1.8
Killip class II/III/IV	25.2	15.1	29.4	<0.001	−29.6 *	19.1	19.3	19.1	0.961	0.4
LVEF < 40%	23.3	19.4	24.8	0.05	−14.3 *	18.8	19.3	18.8	0.876	1.2
CVD	38.8	35.7	40.1	0.086	−5.2	35.4	34.8	35.4	0.876	−1.3
Cardiogenic shock	2.8	4.4	2.1	0.007	20.3 *	5.2	5.7	5.2	0.777	2.8
Renal failure	9.8	6.8	11.0	0.006	−11.3 *	8.3	8.1	8.3	0.921	−0.8
Prior PCI/CABG	28.5	31.6	27.2	0.06	17.3 *	32.0	32.1	31.9	0.969	0.3
**In-hospital medications,** n (%)										
UFH/LMWH	85.9	83.9	86.8	0.116	−11.5 *	84.7	84.5	84.7	0.93	−0.8
GP IIb/IIIa blocker	5.6	15.5	1.5	<0.001	56.3 *	15.7	17.9	15.6	0.462	7.9
Thrombolytic	0.2	0	0.2	0.267	−8.7	0	0	0	1.000	0
Inotropes	5.4	8.5	4.1	<0.001	27.4 *	9.8	10.8	9.7	0.666	4.2
**Medications at discharge,** n (%)										
Aspirin	92.1	98.5	89.5	<0.001	32.8 *	98.3	98.3	98.2	0.965	0.2
Platelet inhibitor	70.9	93.8	61.5	<0.001	71.4 *	91.7	91.9	91.7	0.921	0.6
RAS blocker	74.7	73.5	77.7	0.062	6.4	78.2	78.4	78.1	0.941	0.6
Beta blocker	79.9	85.8	77.5	<0.001	22.7 *	86.1	86.1	86.1	0.99	0.1
Statin	93.0	98.1	90.9	<0.001	30.6 *	98.6	98.6	98.6	0.969	0.2

EB, entropy-balancing matching method; PCI, percutaneous coronary intervention; CABG, coronary artery bypass graft; SMD, standardized mean difference (%); BMI, body mass index (n = 1806); Trop, troponin (n = 1778); GRACE, global registry of acute coronary events (n = 1812); LVEF, left ventricular ejection fraction (n = 1178); CVD, cerebrovascular disease (stroke/transient ischemic attack, coronary artery disease, peripheral vascular disease); UFH, unfractionated heparin; LMWH, low molecular weight heparin; GP, glycoprotein; platelet inhibitors included clopidogrel, ticagrelor, and prasugrel; RAS, renin–angiotensin blocker. * SMD values above 10% are indicative of covariate imbalance, as suggested by Normand and colleagues [17].

**Table 2:** Impact of PCI/CABG on annual follow-up major adverse cardiovascular events (MACE) after the index hospitalization in NSTEMI ACS patients in the Arabian Gulf utilizing the entropy-balancing (EB) matching method.

Outcome Characteristics	Original Unmatched Cohort (n = 1820)			EB Matched Cohort (n = 1050)		
	PCI/CABG			PCI/CABG		
	Without (n = 1291)	With (n = 529)	*p*-Value	Without (n = 762)	With (n = 288)	*p*-Value
**MACE**	563 (44%)	140 (26%)	<0.001	326 (43%)	71 (25%)	<0.001
Stroke/TIA	73 (5.7%)	15 (2.8%)	0.011	53 (7.0%)	7 (2.4%)	0.005
Reinfarction	17 (1.3%)	10 (1.9%)	0.358	11 (1.4%)	5 (1.7%)	0.73
All-cause mortality	184 (14%)	27 (5.1%)	<0.001	100 (13%)	13 (4.5%)	<0.001
Cardiac-related readmission	399 (31%)	112 (21%)	<0.001	235 (31%)	58 (20%)	0.001

PCI, percutaneous coronary intervention; CABG, coronary artery bypass graft; NSTEMI, non-ST-elevation myocardial infarction; ACS, acute coronary events; ATT, average treatment effect on the treated; MACE event included either a stroke/TIA, reinfarction, mortality, or readmission for cardiac reason; CI, confidence intervals; TIA, transient ischemic attack.

**Table 3:** Demographic and clinical characteristics between the cohort remaining at the end of the year and the cohort that was lost to follow-up (LTF).

Characteristic	LTF (n = 68) 3.7%	Remaining (n = 1752) 96.3%	*p*-Value
**Demographic, mean ± SD unless stated otherwise**			
Age, years	62.1 ± 12.8	61.7 ± 12.2	0.788
Male gender, n (%)	48 (70.6%)	1142 (65.2%)	0.358
BMI, kg/m^2^	28.5 ± 5.1	28.5 ± 5.8	0.946
Smoker, n (%)	22 (32.4%)	355 (20.3%)	0.011
**Clinical, mean ± SD or median (IQR) unless stated otherwise**			
GRACE risk score	135.3 ± 44.1	132.0 ± 38.2	0.491
Atrial fibrillation, n (%)	3 (4.4%)	81 (4.6%)	0.935
Troponin, ng/ml	0.2 (0.1–1.8)	0.3 (0.1–2.9)	0.552
Diabetes mellitus, n (%)	39 (57.4%)	1009 (57.6%)	0.969
Hypertension, n (%)	41 (60.3%)	1242 (70.9%)	0.06
Dyslipidemia, n (%)	35 (51.5%)	1052 (60.1%)	0.157
Killip class II/III/IV, n (%)	15 (22.1%)	444 (25.3%)	0.541
LVEF <40%, n (%)	6 (12.2%)	269 (23.8%)	0.061
CVD, n (%)	27 (39.7%)	679 (38.8%)	0.875
Cardiogenic shock, n (%)	3 (4.4%)	47 (2.7%)	0.392
Renal failure, n (%)	8 (11.8%)	170 (9.7%)	0.574
Prior PCI/CABG, n (%)	24 (35.3%)	494 (28.2%)	0.203
**In-hospital medications, n (%)**			
UFH/LMWH	62 (91.2%)	1502 (85.7%)	0.205
GP IIb/IIIa inhibitor	2 (2.9%)	99 (5.7%)	0.338
Thrombolytic	0	3 (0.2%)	0.733
Inotropes	5 (7.4%)	93 (5.3%)	0.464
**Medications at discharge, n (%)**			
Aspirin	55 (80.9%)	1621 (92.5%)	<0.001
Platelet inhibitor	43 (63.2%)	1247 (71.2%)	0.157
RAS blocker	45 (66.2%)	1315 (75.1%)	0.098
Beta blocker	54 (79.4%)	1400 (79.9%)	0.92
Statin, n (%)	60 (88.2%)	1632 (93.2%)	0.12

SD, standard deviation; BMI, body mass index; IQR, interquartile range; LVEF, left ventricular ejection fraction; CVD, cerebrovascular disease (stroke/transient ischemic attack, coronary artery disease, peripheral vascular disease); PCI, percutaneous coronary intervention; CABG, coronary artery bypass graft; UFH, unfractionated heparin; LMWH, low molecular weight heparin; GP, glycoprotein; platelet inhibitors included clopidogrel, ticagrelor and prasugrel; RAS, renin-angiotensin blocker. Percentages may not add up too 100% due to rounding off.

## Data Availability

Data were anonymized for this study. All data are available upon request to the corresponding author.
